# Plantar Fasciitis Research: A Bibliometric Analysis From 2010–2024

**DOI:** 10.1002/jfa2.70136

**Published:** 2026-02-27

**Authors:** Baoqiang Xu, Guanghui Zhang, Zhi Zhang, Lei Zhang

**Affiliations:** ^1^ Hand and Microvascular Surgery Affiliated Hospital of Jining Medical University Jining City Shandong Province China

**Keywords:** bibliometric, extracorporeal shock wave therapy, heel pain, plantar fasciitis

## Abstract

**Objective:**

Plantar fasciitis is a common degenerative foot disease that significantly impairs quality of life. The disease is characterized by multifactorial pathogenesis, diverse intervention strategies, and heterogeneous therapeutic effects. Despite extensive research findings, the fragmented nature of these results hinders a comprehensive understanding of the field.

**Methods:**

This study employed a bibliometric approach to analyze the literature data obtained from the Web of Science database over the past 15 years. The aim was to explore the knowledge structure, research trends, and collaborative features of this field from a quantitative perspective using bibliometric analysis.

**Results:**

The study revealed a fluctuating trend in publications within the field, with the United States, Harvard Medical School, and Karl B. Landorf leading the research output and collaboration. *Foot and Ankle International* emerged as the most prolific and frequently cited journal in this domain. The research hotspots in this field primarily focus on “plantar fasciitis,” “heel pain,” and “extracorporeal shock wave therapy.” Meanwhile, “shear wave elastography,” “plantar fascia thickness,” “systematic review,” and “musculoskeletal disease” represent the research trends in this field. In addition, this study identifies the literature that has had a significant impact on the field.

**Conclusion:**

By organizing the entire research field of plantar fasciitis, this study provides decision support for future clinical practice and scientific research.

## Graphical abstract

Based on bibliometric analysis, this study systematically reviewed the literature on plantar fasciitis from the past 15 years. The findings revealed publishing trends, identified key scholars, authoritative institutions, and influential countries. Additionally, the study clarified research hotspots and predicted future research directions. This provides a detailed roadmap for scientific research exploration and clinical practice in this domain.

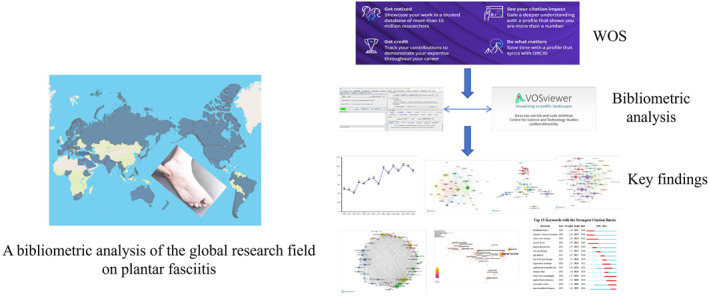

**Abbreviations**
EWST: extracorporeal shock wave therapy
PF: plantar fasciitis
PRP: platelet‐rich plasma

## 1 Introduction

Plantar fasciitis (PF) is a common musculoskeletal disorder and one of the leading causes of heel pain, particularly in adults aged 45–64 years [1]. From different perspectives, some scholars have referred to it as plantar heel pain, plantar fasciosis, or plantar fasciopathy [2, 3, 4]. Based on the results of the literature retrieval, the term “plantar fasciitis” was used most frequently. PF typically presents as severe heel pain upon waking in the morning or after prolonged periods of rest. Although activity may alleviate pain, standing or walking for long durations can worsen it [5]. Research indicates that the causes of PF are multifactorial [6]. Common intrinsic factors include anatomical foot abnormalities, body mass index, age, and sex, whereas extrinsic factors include physical activity levels, occupational exposure, and other systemic diseases are extrinsic factors [7, 8, 9]. Conservative treatment, including podiatry, physiotherapy, and pharmacological agents, is preferred for PF. Podiatry and physiotherapy modalities, such as plantar fascia and gastrocnemius muscle stretching, custom foot orthoses, and extracorporeal shock wave therapy, have demonstrated significant clinical efficacy [10, 11, 12]. The pharmacological treatment of the disease mainly focuses on the use of oral NSAIDs and local injections of corticosteroids or other preparations. Local injections appear to be more effective than other delivery methods [13]. When conservative treatment is ineffective, surgical intervention may become the final option [14]. PF treatment should follow an individualized treatment regimen, and further observation and research are required to ascertain the long‐term effects of various available treatment options [15].

Early studies on PF concentrated on its anatomy and pathology, with current research extending to its etiology, diagnosis, treatment, and prevention strategies. However, a systematic analysis of the research status, trends, and hotspots in this field is lacking. Bibliometric is a quantitative analysis of the characteristics of published literature and is used to describe, evaluate, or predict the research process and development trends in a certain field. This study adopted a bibliometric method to systematically analyze the development trends of scientific research in the field of PF. The aim of this study was to comprehensively describe the regularity and connection of PF fields.

## 2 Method

### 2.1 Database and Data Retrieval

**FIGURE 1:**
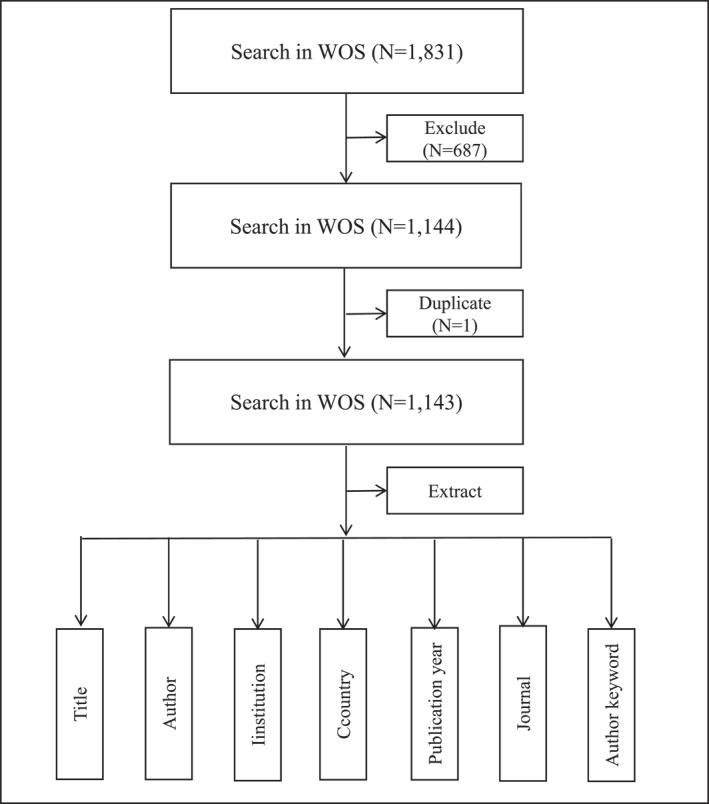
Data retrieval and extraction flowchart.

The data for this study were obtained from the Web of Science (WOS) Core Collection, which is a comprehensive and widely recognized bibliographic database. A free‐term search was conducted using the keyword “plantar fasciitis” in the topic field, and 1831 articles were obtained. The inclusion criteria include: (1) The paper must be published between 2010 and 2024; (2) it should be an article or a review article; and (3) the language of the paper must be English. The exclusion criteria include: (1) Duplicate paper; (2) document types are book chapters, proceeding papers, early access, or retracted publication; and (3) the language was not English, and the publication year was not within the specified range. According to the inclusion and exclusion criteria, 1143 papers were included as shown in Figure 1.

### 2.2 Tool and Analysis

The records were exported to a “Plain Text File.” Select “Full Record and Cited References” for the record content. VOSviewer (version 1.6.13) and CiteSpace (6.2.R3) were used to open the exported papers and extract relevant data, including the title, author, institution, country, journal, year of publication, and author keywords. Data extraction was independently completed by two researchers and confirmed after a joint discussion. In addition, some data are directly quoted from databases, such as the H‐index. This was followed by bibliometric analysis, such as collaborative network, co‐citation, clustering, and burst (the frequency increases suddenly in a certain period) analysis (Figure 1).

## 3 Results

### 3.1 Data Summarization

A summary of the extracted literature data is presented in Table 1. A total of 4678 authors published papers in this field, with papers published in 332 journals. Over the past 15 years, these studies have used 1923 different keywords. Collectively, these publications have cited 10,819 references, received 22,461 citations, and had an H‐index of 65, reflecting their academic influence in this field.

**TABLE 1:** An overview of data on PF from the past 15 years.

Item	Number
Journal	332
Author	4678
Keyword	1923
Reference	10,819
Times cited	22,461
H‐index	65

### 3.2 Annual Number of Publications

**FIGURE 2:**
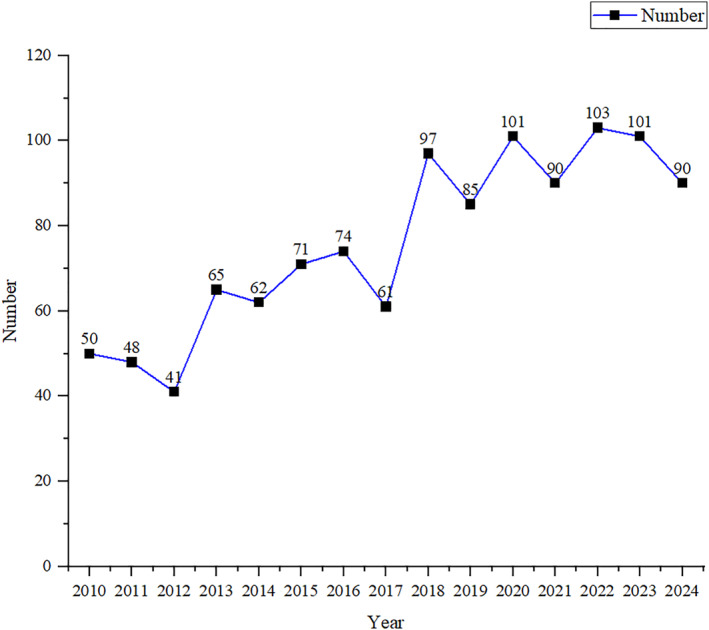
Annual number of publications on PF over the last 15 years.

A line chart of the annual number of publications in the PF field over the past 15 years is presented in Figure 2. Overall, the number of publications in the PF field showed a fluctuating growth trend. Between 2010 and 2012, 139 articles were published, with an average of approximately 46 papers per year. In the 5 years from 2013 to 2017, the total number of papers published increased to 333, an average of approximately 66 per year, representing a growth rate of 43.4% compared with that of the previous stage. From 2018 to 2024, the total number of annual publications reached 577, with an average of 95 papers per year, an increase of 43.9% compared to that of the previous period. Over the past 15 years, research in the field of PF has focused on two topics: “heel pain” and “extracorporeal shock wave therapy,” highlighting their continued importance in the field.

### 3.3 Cooperative Relationship Analysis

**FIGURE 3:**
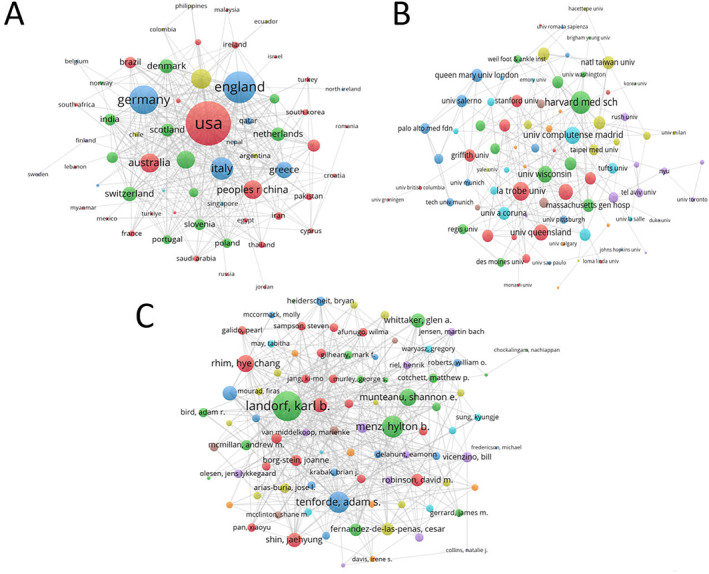
Map of cooperative relationships (node size represents the strength of collaboration, color represents clusters, and line thickness represents the frequency of collaboration). (A) National cooperation, (B) institutional cooperation, and (C) author cooperation.

A total of 68 countries, 1808 institutions, and 4678 authors contributed to the publications in this field. Table 2 presents the top 10 countries, institutions, and authors by the number of publications. Collaboration networks among countries, institutions, and authors were visualized using VOSviewer (Figure 3), where the size of the nodes reflects the frequency of collaboration, and the thickness of the lines indicates the strength of the partnership. As shown in Table 2 and Figure 3, the United States (*n* = 356) had the largest number of articles published in the PF field, followed by China (*n* = 147). Harvard Medical School was the most prolific institution (*n* = 29). Dr. Karl B. Landorf was the most prolific scholar (*n* = 16). Notably, these subjects also possessed the largest nodes in the cooperative network map, indicating a broader range of cooperation.

**TABLE 2:** Top 10 countries, institutions, and authors by number of publications (information in one row does not correspond).

Country			Institution			Author		
Rank	Name	Count	Rank	Name	Count	Rank	Name	Count
1	USA	356	1	Harvard Med Sch	29	1	Landorf, Karl B.	16
2	China	147	2	La Trobe Univ	26	2	Tenforde, Adam S.	14
3	England	78	3	Hong Kong Polytech Univ	13	3	Menz, Hylton B.	11
4	Turkey	76	4	Chang Gung Univ	12	4	Munteanu, Shannon E.	8
5	Spain	67	5	Harvard Univ	12	5	Fleischer, Adam E.	7
6	Australia	60	6	Natl Taiwan Univ	12	6	Whittaker, Glen A.	7
7	Germany	59	7	Rush Univ	12	7	Bonanno, Daniel R.	6
8	Italy	53	8	Univ Michigan	12	8	Fernandez‐de‐las‐Penas, Cesar.	6
9	South Korea	53	9	Univ Seville	12	9	Cleland, Joshua A.	5
10	Brazil	38	10	Univ Wisconsin	12	10	Cotchett, Matthew P.	5

### 3.4 Journal Publication Analysis and Citation Network

**FIGURE 4:**
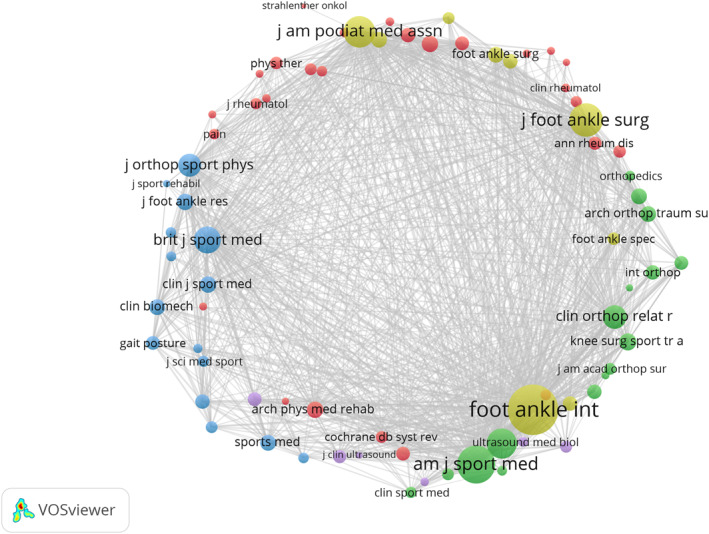
Journal co‐cited network map (the size of the nodes represents co‐cited strength, the thickness of the lines represents co‐cited frequency, and the color represents clustering).

Table 3 lists the top 10 journals ranked by the number of publications along with their key characteristics. A co‐citation network map of the journals was created using VOSviewer (Figure 4). In this map, the size of each node indicates the total co‐citation frequency and the lines represent the co‐citation intensity between nodes. *Foot and Ankle International* published the highest number of papers in this field, with a total of 73. The *Journal of Foot and Ankle Surgery* was the second most productive journal, with 70 papers published. Furthermore, the corresponding node of *Foot and Ankle International* is the most prominent node in the graph, indicating that it has the highest citation frequency and revealing its influence in the PF field. The most frequently cited paper from *Foot and Ankle International* is titled “Relationship Between Tightness of the Posterior Muscles of the Lower Limb and Plantar Fasciitis” [16].

**TABLE 3:** Top 10 journals in terms of publications.

Rank	Journal	Count	Country	JCR (2024)	IF (2024)
1	*Foot and Ankle International*	73	USA	Q2	2.2
2	*Journal Of Foot and Ankle Surgery*	70	USA	Q3	1.3
3	*Journal Of The American Podiatric Medical Association*	47	USA	Q4	0.5
4	*Journal Of Foot And Ankle Research*	33	England	Q1	2.2
5	*Foot And Ankle Surgery*	30	England	Q2	1.9
6	*Medicine*	19	USA	Q2	1.4
7	*Pm&R*	15	USA	Q2	2.8
8	*Bmc Musculoskeletal Disorders*	14	England	Q3	2.4
9	*Foot And Ankle Clinics*	13	USA	Q3	1.6
10	*PloS One*	13	USA	Q1	2.6

### 3.5 Co‐Cited Analysis of Literature

**FIGURE 5:**
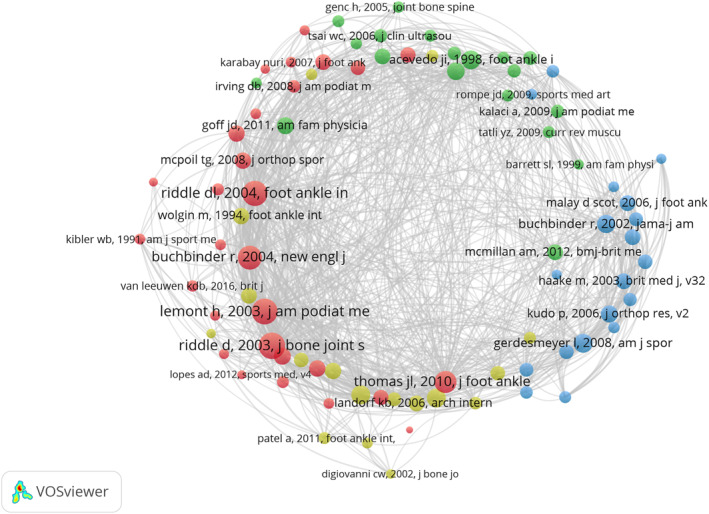
Literature co‐cited network map (the size of the nodes represents co‐cited strength, the thickness of the lines represents co‐cited frequency, and the color represents clustering).

**FIGURE 6:**
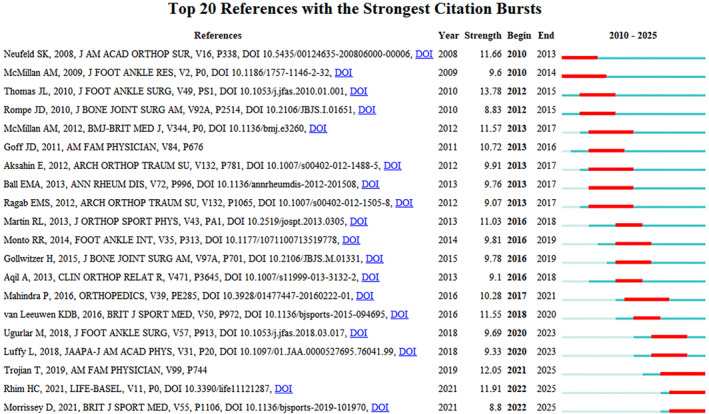
Burst map of the co‐cited literature (the short horizontal line represents the time period of appearance. The darker the color, the higher the frequency of appearance. Red represents the time period when high‐frequency words appear intensively).

The literature co‐citation network was visualized using VOSviewer (Figure 5), and the literature was analyzed for bursts using CiteSpace (Figure 6). The bursts refers to the sudden and frequent occurrence of certain words within a specific period of time. The following is the same as above. The largest node in Figure 5 corresponds to the paper titled “Risk Factors for Plantar Fasciitis: A Matched Case‐Control Study” [17]. As shown in Figure 6, the top 20 references with the strongest citation bursts were identified using the CiteSpace software. The paper with the strongest burst strength was “The Diagnosis and Treatment of Heel Pain: A Clinical Practice Guideline–Revision 2010.” [18]. This paper was published in 2010 in the *Journal of Foot and Ankle Surgery*, with a burst intensity value of 13.78 and a burst duration of 4 years from 2012 to 2015. This paper serves as a clinical guide for the etiology, diagnosis, and treatment of heel pain. The tertiary treatment plan for heel pain proposed in this study is the focus of academia and has an important influence in the field. Furthermore, Figure 6 shows that three papers have burst durations extending to 2024. The paper entitled “Plantar Fasciitis” [19]undertakes a review of the etiology, risk factors, diagnosis, imaging, and treatment of PF and indicates that conservative treatment is the preferred treatment for PF. A paper entitled “A Systematic Review of Systematic Reviews on the Epidemiology, Evaluation, and Treatment of Plantar Fasciitis” by Rhim et al. [20] also showed a burst from 2022 to 2024. According to this study, body mass index is the primary risk factor for PF, with clinical evaluation and physical examination serving as the diagnostic criteria and ultrasound being the most effective imaging technique. Although extracorporeal shock wave therapy (ESWT) and platelet‐rich plasma (PRP) are more effective, the optimal treatment regimen requires further exploration. Another paper is “Management of plantar heel pain: a best practice guide informed by a systematic review, expert clinical reasoning and patient values” [21]. This study adopted a mixed research approach to provide clinical practice guidelines for patients experiencing plantar heel pain.

### 3.6 Keyword Analysis

#### 3.6.1 Keyword Co‐Occurrence and Cluster

**FIGURE 7:**
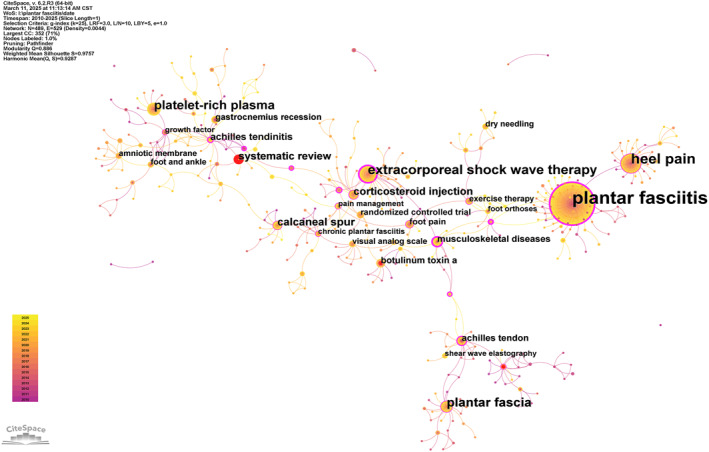
Keyword co‐occurrence map (node size represents the total frequency of occurrence, whereas the connecting lines represent the frequency of co‐occurrence between the two.).

**FIGURE 8:**
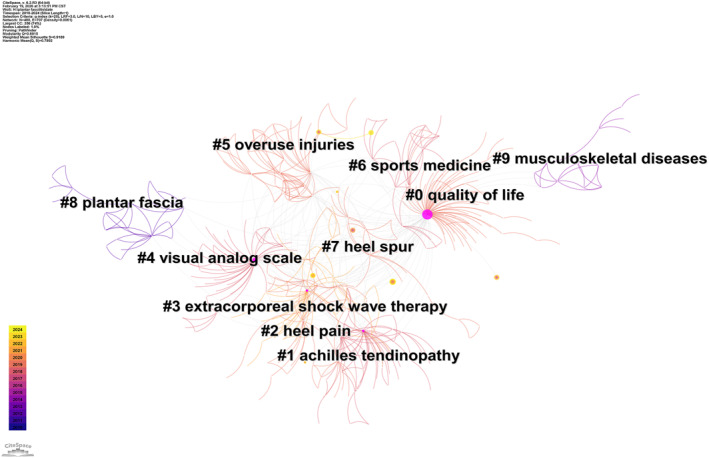
Keyword cluster map.

Keywords, particularly author‐defined terms, summarize the core content of scientific publications. The co‐occurrence of keywords refers to their simultaneous appearance in the same article, with network map analysis revealing the correlation and hierarchical structure between different research topics. A keyword co‐occurrence network of PF‐related publications from 2010 to 2024 was constructed using CiteSpace (Figure 7). In this network, the size of each node corresponds to the keyword frequency, and the connecting lines indicate the co‐occurrence strength. Upon analyzing the network, the following keywords were identified as the most frequent (in descending order): plantar fasciitis (431), heel pain (118), extracorporeal shock wave therapy (80), plantar fascia (66), platelet‐rich plasma (55), calcaneal spur (30), corticosteroid injection (29), systematic review (28), Achilles tendinitis (20), and botulinum toxin A (16). The analysis of keyword co‐occurrence identified three predominant associations with PF: heel pain (84 co‐occurrences), extracorporeal shock wave therapy (45 co‐occurrences), and platelet‐rich plasma (36 co‐occurrences). As illustrated in Figure 7, these closely interconnected nodes form a central framework and represent the current research hotspots in PF. Keyword clustering refers to the method of classifying highly correlated keywords into one category through statistical analysis of keyword co‐occurrence relationships. Core themes in a specific area can be identified using keyword clustering analysis. Figure 8 presents the top 10 keyword clusters and their respective topics in detail.

The first clustering (#0) theme was quality of life, which contained 58 keywords. Representative keywords included plantar fasciitis, extracorporeal shock wave therapy, plantar pressure, and fasciitis plantar. The theme of the second cluster (#1) was Achilles tendinopathy, featuring keywords such as foot and ankle, growth factors, ankle equinus, and autologous blood. The third cluster (#2) focused on heel pain. Other keywords included the foot function index, plantar heel pain, and platelet‐rich plasma. The label for Cluster 4 (#3) was extracorporeal shock wave therapy. Other keywords included carpal tunnel syndrome, chronic heel pain, endoscopic plantar fasciotomy, and photobiomodulation therapy. The visual analog scale (VAS) was the label for the fifth cluster (#4). Additional keywords were as follows: botulinum toxin A, chronic pain, randomized controlled trial, and botulinum toxin. The theme of the sixth cluster (#5) was overuse injuries. Other keywords included magnetic resonance imaging, running injuries, and stress fractures. The theme of the seventh cluster (#6) was sports medicine. Other keywords included ankle sprains, systematic reviews, and musculoskeletal pain. The theme of the eighth cluster (#7) was heel spur. Other keywords included chronic plantar fasciitis, benign degenerative disease, treatment outcomes, and plantar fascia thickness. The ninth cluster (#8) was characterized by plantar fasciitis. Additional keywords included plantar aponeurosis, Baxter nerve, windlass effect, and windlass mechanism. The theme of the 10th cluster (#9) was musculoskeletal diseases. Other keywords included Achilles tendon, musculoskeletal system, gastrocnemius tightness, and gastrocnemius muscle.

#### 3.6.2 Research Hotspot Changes and Research Trend Analysis

**FIGURE 9:**
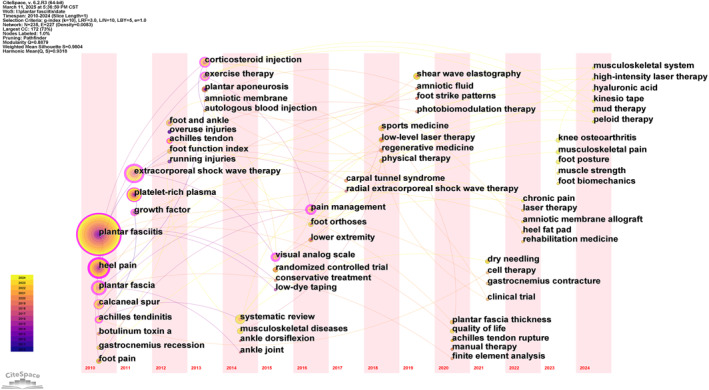
Keyword evolution time‐zone map (the node position indicates the year of initial occurrence, the node size reflects the cumulative frequency of occurrence, and the connecting lines represent the co‐occurrence frequency).

In order to explore the changes in research topics in the field of PF, this study used CiteSpace software to draw the time zone map of keywords as shown in Figure 9. The position of each node in the figure indicates the year in which the corresponding keyword first appeared within the search time range. The meaning of the nodes and connecting lines was consistent with that of the keyword co‐occurrence mapping. Keywords, such as plantar fasciitis, heel pain, plantar fascia, and calcaneal spur, first appeared in 2010 and continued to receive significant attention over the following 15 years. From 2011 to 2012, treatment methods became a focal point, with emerging therapies, such as extracorporeal shock wave therapy, platelet‐rich plasma, and growth factors, appearing sequentially. From 2011 to 2012, the focus shifted to treatment methods, with innovative therapies, such as extracorporeal shock wave therapy, platelet‐rich plasma, and growth factors, introduced consecutively. Between 2013 and 2014, the focus shifted to corticosteroid injections, exercise therapy, systematic reviews, and musculoskeletal disorders. From 2015 to 2016, keywords, such as visual analog scale, randomized controlled trials, and pain management, were first mentioned and received significant attention. Notably, keywords that appeared after 2017 are shown as small nodes on the map, which may be related to their later appearance.

**FIGURE 10:**
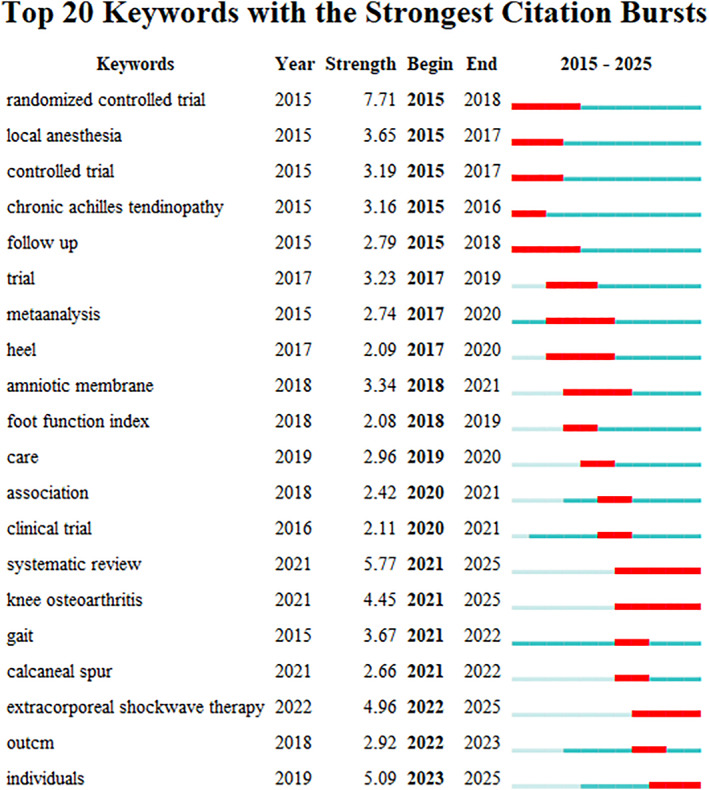
Keywords burst map (he short horizontal line represents the time period of appearance. The darker the color, the higher the frequency of appearance. Red represents the time period when high‐frequency words appear intensively).

A keyword burst refers to a significant increase in the frequency of detected keywords over a specific period. It can identify emerging research focuses and aid in predicting research trends. A keyword burst map was constructed using CiteSpace (Figure 10). The map shows the top 15 keywords with the strongest emergence intensity (an algorithm specifically designed to calculate the rate of change in time series data), along with when they appeared and the years when they began and ended. According to Figure 9, the keywords “shock wave therapy” and “shear wave elastography” have the longest emergence duration (the duration of a sudden high‐frequency occurrence), both lasting for 5 years. This indicates that they have garnered sustained attention and may continue to be research hotspots in the future. Keywords, such as “shear wave elastography,” “plantar fascia thickness,” “systematic review,” and “musculoskeletal disease,” began to burst in different years and continued until 2024. These represent new concerns and research trends in the field. Among them, the intensity of “systematic review” was as high as 5.33, indicating that this research method has received considerable attention from the academic community and is a crucial tool in the field of PF research.

## 4 Discussion

This study employed bibliometric methods to explore the profile of the entire PF research field by visualizing and analyzing literature data. It is used to sort out the context and framework of the entire related topic. The research content of this study mainly focuses on three aspects: publishing trends, contributors, and hotspots.

Over the past 15 years, there has been a fluctuating upward trend in the number of PF publications. The cumulative number of published papers is also increasing. Every progress, breakthrough, or innovation in the field of PF has led to a significant increase in research output, such as “corticosteroid injection,” “autologous blood injection,” and “low‐level laser therapy,” proposed in the past 15 years [22, 23, 24]. With the global population aging, the incidence rate of 7% in the elderly over 65 years old leads to a continuous rise in the overall prevalence of the disease, drawing sustained attention from the academic community [25]. Notably, PF is controversial in many respects, including naming, treatment, and prognosis, which is also the driving force behind the increase in research output and sustained attention.

The United States, China, the United Kingdom, and Turkey are leaders in PF research, but their research directions and cooperation objects differ. The research in the United States mainly focused on the direction of “Platelet‐Rich Plasma,” and the number of related papers reached 53 [26]. The United States collaborates most frequently with Germany, reaching 19 times. Harvard Medical School is well‐known among American institutions for its PF research. China ranked second in the number of publications, with a focus on “extracorporeal shock wave therapy” [27]. The United States is China’s closest partner (7 times). Professor Karl B. Landorf from La Trobe University has conducted in‐depth research on PF, exploring the vital role of ultrasound technology in diagnosing and treating the condition [28]. In addition, the “foot orthoses” is also an important research direction of Professor Karl B. Landorf in the field of PF [29, 30]. The analysis of PF contributors revealed the shortcomings of the research in this field: (1) the geographic distribution is too concentrated, with seven of the top 10 countries by publication volume being concentrated in Europe and the United States; and (2) current collaboration models tend to be infrequent and have a limited scope, usually involving only a few researchers within the same institution. These limitations have become major constraints that hinder the development of this field.

Keywords analysis of the published literature on PF revealed that research hotspots were concentrated primarily on disease treatment. The most representative of these treatments were corticosteroid injection, extracorporeal shockwave therapy (ESWT), and platelet‐rich plasma (PRP). Corticosteroid injection for PF has a short duration of effect, with long‐term efficacy at 8–12 weeks no better than placebo [31]. Repeated corticosteroid injections increase the risk of complications. The American Orthopedic Foot and Ankle Society (AOFAS) reported a 4% chance of skin atrophy and a 1.4% chance of heel fat pad atrophy [32]. Compared with corticosteroid injection, ESWT has more advantages in relieving pain, improving foot function, and reducing plantar fascia thickness and its efficacy is more significant in the medium and long term [33, 34, 35]. In addition, ESWT has some advantages in reducing plantar fascia thickness [34]. Studies have shown that PRP injections are better than corticosteroid injections for pain control in the middle term (3–6 months) [36, 37]. In some cases, PRP is superior to ESWT in terms of the VAS pain score and foot function index; however, PRP requires more advanced operating techniques [38, 39]. Although the above three treatment methods have attracted much attention from scholars, they are only applicable to patients who have failed conservative treatment in clinical practice.

To date, no bibliometric analysis has been conducted on PF. However, it is limited by certain conditions when conducting research on it. First, choosing high‐frequency words in the literature as search terms among many controversial keywords and conducting data retrieval in only one database may result in some documents not being identified. However, considering the large number of included studies, this limitation may have little effect on the final results. Second, the cross‐use of two bibliometric software increases the perspective and comprehensiveness of the analysis, but it still cannot cover the shortcomings of quantitative data research. Finally, our analysis was limited to papers from a specific time period, giving the study a sense of urgency. Despite these effects, it does not affect the “pioneering” value of this study and the significance of PF’s overall domain analysis of PF.

This study employed bibliometric methods to systematically analyze the state of research in the PF field. Key contributors, influential journals, and important publications in the field were identified, and research hotspots and future trends were sorted. This study not only addresses existing gaps but also provides a theoretical foundation and data support for subsequent research.

## Author Contributions

**Baoqiang Xu:** conceptualization, validation, visualization, writing – original draft preparation, writing – review and editing. **Guanghui Zhang:** data curation, investigation, project administration, validation, writing – review and editing. **Zhi Zhang:** formal analysis, methodology, software, supervision, writing – review and editing. **Lei Zhang:** funding acquisition, resources, validation, writing – review and editing.

## Funding

Jining Medical University 2024 Annual Practical Teaching Education Research Program Project (Project No.: JYSJ2024C05): Construction of a Skills Teaching and Evaluation System for Undergraduate Clinical Medicine Based on Post Competency—Orthopedic Teaching Practice.

## Ethics Statement

The authors have nothing to report.

## Consent

The authors have nothing to report.

## Conflicts of Interest

The authors declare no conflicts of interest.

## Data Availability

The data supporting the findings of this study are available from the corresponding author upon reasonable request.
